# Atypically diffuse functional connectivity between caudate nuclei and cerebral cortex in autism

**DOI:** 10.1186/1744-9081-2-34

**Published:** 2006-10-16

**Authors:** Katherine C Turner, Leonard Frost, David Linsenbardt, John R McIlroy, Ralph-Axel Müller

**Affiliations:** 1 Brain Development Imaging Laboratory, Department of Psychology, San Diego State University, San Diego, CA 92120, USA; 2 Department of Cognitive Science, University of California, San Diego, CA 92093, USA

## Abstract

**Background:**

Autism is a neurodevelopmental disorder affecting sociocommunicative behavior, but also sensorimotor skill learning, oculomotor control, and executive functioning. Some of these impairments may be related to abnormalities of the caudate nuclei, which have been reported for autism.

**Methods:**

Our sample was comprised of 8 high-functioning males with autism and 8 handedness, sex, and age-matched controls. Subjects underwent functional MRI scanning during performance on simple visuomotor coordination tasks. Functional connectivity MRI (fcMRI) effects were identified as interregional blood oxygenation level dependent (BOLD) signal cross-correlation, using the caudate nuclei as seed volumes.

**Results:**

In the control group, fcMRI effects were found in circuits with known participation of the caudate nuclei (associative, orbitofrontal, oculomotor, motor circuits). Although in the autism group fcMRI effects within these circuits were less pronounced or absent, autistic subjects showed diffusely increased connectivity mostly in pericentral regions, but also in brain areas outside expected anatomical circuits (such as visual cortex).

**Conclusion:**

These atypical connectivity patterns may be linked to developmental brain growth disturbances recently reported in autism and suggest inefficiently organized functional connectivity between caudate nuclei and cerebral cortex, potentially accounting for stereotypic behaviors and executive impairments.

## Background

Autism is a neurodevelopmental disorder affecting social, cognitive, linguistic and sensorimotor abilities. These qualitative deficits are pervasive and long lasting. While genetic factors are known to be strong [1], consistent neurological markers for the disorder remain to be fully established. Behavioral markers identifying deficits in sensorimotor processing and social skills are apparent as early as one year of age [2,3]. Sensorimotor deficits include fine motor apraxia [4-6], reduced postural control [7,8], and impaired imitation [9]. Individuals with autism also have delays in language development [10], impaired attention [11], as well as deficits in executive cognitive processes [12,13]. These developmental abnormalities are often discovered during the preschool years. Among the brain areas suspected to be involved in both sensorimotor and cognitive deficits are the caudate nuclei [14-16].

Several types of abnormalities of the caudate nuclei have been noted in autism. Reduced correlation of resting cerebral glucose metabolic rates between the caudate and frontal regions has been seen in children with autism [17]. Sears et al. [18] and Hollander et al. [14] found that enlargement of the caudate nuclei was associated with stereotyped behaviors in autism. Conversely, a cluster-based analysis of structural MRI scans [19] showed that reduced caudate size was correlated with greater impairment on the Childhood Autism Rating Scale (CARS), including deficits in a wide range of abilities such as body movement. While these anatomical findings appear inconsistent, they nonetheless indicate that autism may be associated with volumetric abnormalities of the caudate nuclei. Furthermore, Singh [20] noted serum antibodies to the caudate in children with autism that were not found in typical children. These antibodies may implicate the caudate nuclei in a type of autoimmune dysfunction associated with autism. Related dysfunction of the caudate nuclei may secondarily affect regions that are anatomically connected to these nuclei.

Retrograde transneural transport of the herpes simplex virus has illuminated the anatomical connectivity between caudate nuclei and other brain regions [21,22]. These connections are widespread. The caudate nuclei primarily receive input from frontal, temporal, inferior parietal, pre-occipital, and limbic areas including the amygdala, hippocampus and parahippocampal cortex. The various connections between the caudate and other brain regions have been segregated into circuits [21,22]. Anatomical circuits directly associated with the caudate are the associative, lateral orbitofrontal, and occulomotor circuits. The associative circuit connects the dorsolateral prefrontal cortex with the ventral caudate, and this circuit is believed to regulate executive functions in the brain by unifying cognitive processes such as attention, planning and decision-making [21,23]. Within this circuit, the caudate nuclei may also aid in the selection of rules by which decisions and plans are made and by enhancing working memory [24-26]. The lateral orbitofrontal circuitry, believed to support set switching and inhibition, extends from Brodmann's areas 10 and 12 to the ventromedial caudate [21,22,27,28]. The caudate nuclei send return projections to areas 10 and 12 via the thalamus. Evidence from human and non-human primate studies show that disruptions to the circuitry at the orbitofrontal level result in deficits in short term memory for objects and the processing of stimulus reinforcement contingencies [29,30]. The third loop involving the caudate and the frontal eye fields (FEF) is the occulomotor circuit and is thought to be involved in saccadic eye movements [31]. The FEF, located in Brodmann's area 6 [32], project to the body of the caudate which then sends projections to the substantia nigra. Although the caudate is not considered part of the motor circuit, it has been shown to contribute to working memory in the planning and selection of motor sequences [33-36]. A disruption in the anatomical connectivity between the caudate and any region in one of the circuits is likely to be reflected in weaker functional connectivity within that circuit.

A number of fMRI studies of autism have shown atypical levels of activation in the caudate nuclei for a variety of tasks such as spatial processing [37], finger tapping [38] and face perception [39]. In each of the above mentioned studies, autism groups showed reduced caudate activation compared to control subjects. These fMRI findings only provide information on whether the caudate nuclei are involved in a task. As established above, however, participation of the caudate nuclei in sensorimotor, cognitive and executive functions reflect their role in distributed functional *networks*. The integrity of networks cannot be fully examined by conventional fMRI activation analyses, which are unsuited for analyzing circuitry.

In order to examine network integrity, the present study used functional connectivity magnetic resonance imaging (fcMRI). Functional connectivity measures examine the temporal covariance between spatially remote neurophysiological events [40]. Previous fcMRI studies have shown that regional signal covariance is consistent with anatomical connectivity and functional networks delineated in animal studies [41,42]. In humans, fcMRI has been used to examine interhemispheric connectivity in the sensory [43,44] and motor cortex [45]. Belmonte et al. [46], Just and colleagues [47] and Sporns et al. [48] have suggested that local connectivity may be relatively dense whereas long-range connectivity between brain regions may be reduced. However, the functional connectivity between basal ganglia and cerebral cortex has not been previously examined in autism. In the present study, we examine functional connectivity of the caudate nuclei in individuals with autism and healthy controls.

## Methods

Participants were eight male autistic patients (mean age: 28.1 years, range: 15–39, SD: 8.3) and eight gender-matched healthy comparison subjects (mean age: 28.6 years, range: 21–43, SD: 7.2). Each patient met the DSM-IV criteria for autism and the criteria for a diagnosis of autism according to the Childhood Autism Rating Scale [49] and the Autism Diagnostic Interview-Revised [50] except for one patient, whose scores fell below the cutoff on the Childhood Autism Rating Scale but were consistent with a diagnosis of autism on the other three measures. All patients had a full-scale IQ above 70, as assessed by the Wechsler Adult Intelligence Scale-Revised or the Wechsler Intelligence Scale for Children-Revised. No additional abnormalities were identified during a neuroradiological examination. Aside from age and gender, groups were also matched for handedness, with three non-right handers per group. Mean performance IQ in the autism group was in the normal range (mean 92.3, range: 80–112). Although we thus ensured that nonverbal domains, which include some relatively spared areas of functioning such as block design [51], were close to the normal, we did not attempt to match groups on verbal or full-scale IQ. Note that the pervasive nature of the disorder implies impairment on many functions tested in a Wechsler IQ test, even non-verbal ones. For example, the extensive literature on executive impairments in autism (see Discussion and ref. [52]) implies that a broad range of cognitive deficits is intrinsic to the autistic condition.

The study was approved by the instititutional review boards of the University of California, San Diego, and San Diego State University. Written consent was obtained from each subject prior to the study.

### Experimental conditions

There were 6 blocks in the experiment, with two conditions (control and experiment) presented in the order ABABAB. Participants viewed the outline of a hand on a screen and performed finger movements with the preferred hand. Responses were prompted by a blue dot appearing on one of the fingers of the hand stimulus on the screen. Instructions were to press as fast as possible the button on a four-button box corresponding to the finger indicated by the dot. In condition A, a blue dot was presented on the index finger every 550 msec. In condition B, pseudo-random 6-digit sequences were presented at the same rate as in the control condition. Within each block, the sequence was repeated 10 times, but a novel sequence was used in each new block of this condition.

### MRI acquisition

Images were acquired on a General Electric Signa 1.5-T scanner (GE Medical Systems, Milwaukee) by using a custom-made head gradient coil. Sagittal and axial localizer scans were used to select sagittal slices for echoplanar image acquisition, ensuring complete coverage of the head. After manual shimming for reduction of magnetic field inhomogeneities, echo planar images were acquired with a single-shot gradient-recalled pulse sequence (interleaved slice acquisition; repetition time [TR] 2500 ms; echo time [TE] 40 ms; flip angle 90°; matrix 64 × 64; field of view [FOV] 24 cm; 19 sagittal slices with a thickness of 7 mm [1 mm gap]; in-plane voxel size 3.75 mm^2^). For each subject, the time series contained 98 echoplanar images. Phase maps were acquired for correction of echoplanar image distortions. A high-resolution structural volume was acquired in the same session, using a three-dimensional magnetization prepared rapid acquisition gradient echo pulse sequence (TR 30 msec; TE 5 msec; flip angle 45°; 256 × 256 × 128 matrix; FOV 24 cm; slice thickness 1.2 mm; in plane voxel size 1 mm^2^).

### FMRI preprocessing and activation analyses

All preprocessing and analyses were performed using the software suite Analysis of Functional NeuroImages (AFNI)[53,54]. Data were corrected for motion, spatially normalized, and spatially smoothed, using a Gaussian filter of 6 mm FWHM (full width at half maximum). Condition B was compared to condition A in terms of hemodynamic changes (reflecting activation [55]). For each participant, time series of signal changes in each voxel (volume element) were regressed against a hemodynamic model (i.e., expected changes for an ideally activated voxel). The model consisted of a boxcar which was smoothed and shifted by 2 TRs, corresponding to an expected hemodynamic latency of 5 sec. Group analyses were performed by means of one-sample *t*-tests, using fit coefficients from individual analyses. Analyses limited to activation effects have been previously published for the present data set [see 56 for detailed description of these methods].

### FcMRI procedures

We applied a functional connectivity procedure similar to those previously used by several groups [41,46]. For each subject, the caudate nuclei were drawn in each hemisphere on the high-resolution anatomical image in native space (i.e., before spatial normalization). These tracings defined regions of interest, which were used as seed volumes, i.e., regions of the brain for which functional connectivity was determined. For each seed volume, a mean time series of all included voxels was computed. Voxel time series were extracted from image sets that had been preprocessed in the following ways: (i) linear trends were removed; (ii) they were temporally smoothed (voxel intensity for time point n = 0.15_n-1 _+ 0.7_n _+ 0.15_n+1_) to remove high-frequency noise; and (iii) they were low-pass filtered at 0.1 Hz. FcMRI effects are known to occur mainly in the low frequency domain below 0.1 Hz [57-59].

Unilateral caudate tracings were combined into a bilateral seed volume in each subject. For each participant, functional connectivity was computed in terms of BOLD signal cross-correlation with the mean time series in the caudate nuclei. The blood oxygenation level dependent (BOLD) signal detected in fMRI is known to reflect indirectly local neuronal activity [55]. Correlation analyses included detected motion in 3 translational axes and 3 rotations as well as a hemodynamic model based on the task-control cycles as orthogonal regressors in order to reduce confounding effects of head motion and activation-related effects of task performance. Fit coefficients were entered into one-sample *t*-tests for groupwise analyses and two-sample *t*-tests for direct group comparisons. Monte-Carlo alpha simulations were used to determine cluster significance at a corrected threshold of *p *< .05 [60]. This procedure takes advantage of the reduced probability of statistical effects occurring in *neighboring *voxels (i.e., in clusters) by chance alone. For within-group analyses, a height threshold of *p *< .00005 (uncorrected) and an extent threshold (i.e., minimum cluster size) of 112 μl were applied. For direct group comparison (two-sample *t*-tests), for which effects were expectedly much less robust, the chosen height threshold was *p *< .01 (uncorrected), with an extent threshold of 344 μl.

In order to address potential confounds related to handedness and lateralization of hemodynamic effects, we also performed analyses for right-handed subsamples, with 5 subjects per group. Due to the lower power in these analyses, a height threshold of *p *< .001 with an extent threshold of 288 μl was used for within-group analyses. For between group analyses, a height threshold of *p *< .01 was used with an extent threshold of 344 μl.

Furthermore, besides analyses for bilateral caudate seeds we also analyzed fcMRI effects for unilateral seeds, using identical thresholds as for the bilateral seed analyses (see Discussion).

## Results

### Behavioral results

There were no significant group differences in performance for condition A (index finger only). In the autism group, the mean number of total button presses was 183.5 (*SD *= 30.1), compared to 169.4 (*SD *= 8.9) in the control group (*p *= .31), with a mean interval between responses of 630 msec (*SD *= 104.1) in the autism group and 664 msec (*SD *= 67.5) in the control group. In condition B, mean reaction times were slower in the autism group (autism: 574.1 msec, *SD *= 179.9; controls: 513.9 msec, *SD *= 81.0). The autism group also made more incorrect button presses per task block (12.2, *SD *= 8.4; or 20.3%), compared to 3.4 (*SD *= 4.3; or 5.7%) in the control group. Group differences were significant for number of errors (*t *(14) = 2.63, *p *< .05), but not for response time (*t *(13) = 0.85, *p *= 0.41).

### Activation results

Findings from conventional activation analyses have been previously described in detail [56] and are summarized here only for background information. Note that activation analyses do not aim at identifying interregional connections, but solely effects driven by a task (compared to a control condition). In both groups, activation effects (i.e., significantly greater hemodynamic signal for condition B, compared to condition A) were observed in frontal and parietal areas, including peaks in bilateral premotor and superior parietal cortex, as well as left postcentral and right inferior frontal gyri. Additional effects in both groups occurred in bilateral temporo-occipital regions and thalamus. Activation peaks in the basal ganglia were seen in the left lentiform nucleus and right putamen for the control group, and on the border of the left putamen and caudate nucleus for the autism group.

### FcMRI results

In both groups, fcMRI effects for the bilateral caudate seed volume were predominantly observed in frontal and parietal lobes.

#### Control group

Significant connectivity with the bilateral caudate seed volumes (Table ' [see additional file 1]') was observed in left middle frontal and medial frontal gyri (Brodmann area [BA] 6), left precentral gyrus (BA 6) and right medial frontal gyrus (BA 8). Effects were also seen in bilateral parietal cortex (BA 40), the cingulate gyri (BA 31 on the left and BA 32 on the right), as well as the precuneus. A few clusters occurred in temporal (BA 22) and occipital regions (BA 19). FcMRI effects associated with the left caudate seed volume (Table 2 ' [see additional file 1]') were observed exclusively in fronto-parietal regions, with clusters in right and left inferior fontal gyri (BA 44, 45), and left middle frontal gyrus (BA 10). Effects for the right caudate seed volumes (Table 3 ' [see additional file 1]') showed a distribution similar to those seen for bilateral seed volumes.

#### Autism group

For the bilateral seed volume (Table 1' [see additional file 1]'), large clusters were observed in the right superior frontal gyrus (BA 8), right precuneus (BA 7) and left posterior cingulate gyrus (BA 31). FcMRI effects were also seen in the left inferior parietal lobe (BA 39/40). For the left caudate seed (Table 2 ' [see additional file 1]'), we observed significant connectivity with frontal areas including right middle frontal gyrus (BA 9) and left medial frontal gyrus (BA 6, 32). Effects were also observed in the precuneus bilaterally (BA 7), in the left superior temporal lobe, and in subcortical regions. Effects for the right caudate seed volume (Table 3 ' [see additional file 1]') were relatively reduced, with only a small number of significant clusters including the right medial frontal gyrus (BA 8), left precentral gyrus (BA 4) and right posterior cingulate gyrus (BA 23).

#### Group comparison

For the bilateral seed volume (Table 1' [see additional file 1]'), direct group comparison revealed a rather diffuse pattern of greater connectivity in the autism group across frontal, parietal, and occipital lobes. Significantly greater fcMRI effects for the autism group were seen in right middle frontal gryus (BA 46), bilateral precentral gyrus (BA 6), left medial frontal gyrus (BA 9), right postcentral gyrus (BA 2, 43), bilateral cingulate gyrus (BA 23), and left cuneus (BA 18). In contrast, only a single region of greater connectivity for the control group was detected in the right superior frontal gyrus (BA 10). Similar to effects for the bilateral seed volumes, group comparisons for the left caudate seed volume (Table 2 ' [see additional file 1]') also showed more areas of connectivity for the autism group compared with the control group in frontal, parietal, and occipital regions. In contrast, for the right caudate seed (Table 3 ' [see additional file 1]') we found predominantly enhanced connectivity for the control group as compared to the autism group, with clusters in the left middle temporal gyrus (BA 21), right parahippocampal gyrus (BA 34), and in occipital cortex (BA 18, 19) bilaterally.

Since the autism group (but not the control group) included one subject under the age of 18 years, we reran the group comparison statistics without this 15-year old subject. Although clusters of group effects tended to be slightly lower with regard to cluster volume and peak t-scores (due to reduced power), regional patterns of effects were virtually identical, with cluster peaks ≤ 2 mm apart.

## Discussion

Neuroanatomical studies have identified a number of functional circuits with participation of the caudate nuclei. The purpose of the present study was twofold: to investigate whether in healthy controls functional connectivity between the caudate and other brain regions reflected the known anatomical connectivity and to examine whether participants with autism would show atypical functional connectivity, given the caudate abnormalities previously observed in autism studies. In within-group analyses for the control group, we found connectivity between bilateral caudate nuclei and frontal, pericentral, parietal, temporal, occipital and subcortical regions, largely consistent with the known participation of the caudate nuclei in several distributed functional networks (as discussed in detail below). In the autism group, connectivity was primarily seen between caudate nuclei and premotor, pericentral and parietal areas. In direct between-group comparisons, fcMRI effects were found to be mostly increased in the autism group relative to the control group. Unilateral caudate seed volumes were examined in addition to the bilateral caudate seeds. In general, group differences for the left caudate seed volume were similar to those for the bilateral seed volume, with greater pericentral effects in the autism group than in the control group. For the right caudate seed volume, however, these group differences were not found and the autism group showed reduced fcMRI effects compared to the control group in occipito-temporal regions.

Based on anatomical circuits that include the caudate nuclei we expected functional connectivity between caudate nuclei and areas found within the associative, the lateral orbitofrontal and the oculomotor circuits [22]. The associative circuit is comprised of the caudate nuclei, other nuclei in the basal ganglia, the thalamus, and dorsolateral prefrontal cortex (DLPFC). DLPFC is known to be involved in executive functions [61-65]. Behavioral evidence suggests that all areas of executive function (including inhibitory control, working memory, planning, and cognitive flexibility) are affected in individuals with autism spectrum disorders [4,52,66,67]. One fMRI study showed reduced activation in DLPFC during executive tasks in individuals with autism [12]. For the bilateral seed volume, we found no effects in DLPFC for either group. However, both groups showed fcMRI effects for the left caudate seed volume in the middle frontal gyrus, with additional inferior frontal clusters in the control group. In the between-group analysis for the bilateral caudate, only a single cluster with greater fcMRI effects in the control than in the autism group was found, located in the frontopolar portion of area 10. Inverse effects (greater in the autism group) were seen in more posterior and in medial portions of the frontal lobe (areas 9, 44, 46). While we did not observe generally reduced fcMRI in DLPFC in our autism sample, the finding of reduced connectivity with right frontopolar cortex was remarkable. Frontopolar area 10 is thought to serve the highest-level executive functions within prefrontal cortex involving integration of multiple supramodal cognitive operations [68].

A further anatomical circuit associated with the caudate nuclei is the lateral orbitofrontal circuit, which includes the lateral orbitofrontal cortex, the superior temporal gyrus (STG), inferior temporal gyrus (ITG), and the anterior cingulate (AC; [22]. No fcMRI effects were seen in the orbitofrontal lobe, which is not surprising given the typically reduced signal in this region on T2*-weighted images. Within-group analyses for the bilateral caudate nuclei showed fcMRI effects for the STG and AC in the control group, which were not seen in the autism group. STG has been shown to play a role in face perception and the detection of gaze [69,70], which are impaired in autism [71-73]. Atypical functional connectivity between caudate nuclei and the anterior cingulate gyri could relate to the known role of the AC in inhibition [74] and resolution of response conflict [75]. However, no firm conclusions with regard to the lateral orbitofrontal circuit can be drawn, since fcMRI effects in the STG were found in the autism group for the left caudate seed and direct between-group statistical comparisons did not yield significant effects in STG or AC for any of the seed volumes.

With regard to the oculomotor circuit, we expected to find functional connectivity between the caudate nuclei and two regions: frontal eye fields (FEF) and supplementary eye fields (SEF) in Brodmann area 6 and posterior parietal cortex [22]. The control group showed fcMRI effects in area 6 for bilateral and unilateral caudate seed volumes, whereas the autism group showed effects in this area only for the left caudate seed. However, in the between-group analysis for bilateral caudate nuclei, greater functional connectivity was observed for the autism group in the superior portion of area 6 in the left hemisphere and in the inferior portion of area 6 in the right hemisphere with Euclidean distances of 30 mm and 26 mm from the location of the FEF and respectively. For the control group, three fcMRI effects in area 6 for bilateral and right caudate seeds were within 10.5 mm of the reported location of the FEF. One fcMRI cluster for the right caudate seed was seen within a Euclidean distance of 3 mm of the SEF [76]. By contrast, most of the abovementioned fcMRI effects for the autism group in area 6 were too distant to be associated with the FEF and SEF. The FEF and SEF are associated with saccadic eye movement [76,77] as well as visual search [78-80]. Autism is characterized by an uneven profile of preserved or enhanced performance on embedded figures and conjunctive search tasks [81-84] accompanied by impairments in visual attention [85]. Fewer fcMRI effects in areas close to the known locations of the FEF and SEF in our autism sample may relate to atypical saccadic eye-movement and visual search abilities [86].

A second region participating in the oculomotor circuit, for which fcMRI effects were expected, was the posterior parietal cortex [22]. Animal studies of caudate connectivity indicate connections to both superior and inferior posterior parietal cortex, including medial portions of the superior parietal lobe [87]. Within-group analyses for the bilateral caudate seed showed fcMRI effects in medial parietal regions, i.e., precuneus and posterior cingulate gyrus, bilaterally for both the control and autism groups. Between-group analyses indicated bilaterally increased fcMRI effects in the posterior cingulate gyrus for autism group, compared to controls. The posterior cingulate gyrus is one of the regions involved in the processing of socially relevant stimuli. Maddock [88,89] found that the posterior cingulate participates in the processing of emotionally evocative stimuli as well as personal memories. There is ample evidence that the ability to process socially salient information is dysfunctional in autism [90]. In a study comparing the perception of familiar (and thus socio-emotionally engaging) faces to unfamiliar faces, Pierce and colleagues [39] found that frontal activity seen in typical adults was absent in autistic subjects, whereas medial parietal activation in precuneus and posterior cingulate was retained. These activation results appear consistent with the present finding of intact functional connectivity between caudate nuclei and medial parietal cortex and may be linked to socio-emotional functions or to additional roles of the posterior cingulate in orienting of attention to a spatial cue [91,92].

Aside from the circuits discussed above, we expected connectivity in the motor circuit, which is anatomically linked to nuclei in close proximity to the caudate nuclei. As mentioned earlier, the caudate is thought to cooperate with the motor system in action planning. Deficits in motor planning and coordination are an established feature of autism [93-96] and are part of the diagnostic criteria [97]. Although neither group showed within-group fcMRI effects in primary motor cortex (Brodmann area 4) for the bilateral caudate seed, the between-group analyses did show greater effects in area 4 for the autism group. FcMRI effects in this area were seen in analyses for unilateral seeds. The control group showed effects in area 4 ipsilateral to the seed on both analyses, whereas the autism group only showed an fcMRI effect in contralateral motor cortex for the right caudate seed. Premotor cortex in Brodmann area 6 showed numerous clusters of fcMRI effects. As described above, some of those areas were in close proximity to the FEF and SEF. However, the remaining effects in area 6 were more likely associated with the motor system. In the within-group analysis for the bilateral caudate seed, such additional fcMRI effects in area 6 were found for the control group, but not the autism group. In the between-group analysis, enhanced functional connectivity for area 6 was found in the autism group for the superior most portion on the left and the inferior portion on the right. Increased functional connectivity between the caudate nuclei and motor cortices seen in the autism group may indicate differences in subcortico-cortical networks recruited during visuomotor coordination and sequence learning in autism, consistent with atypical involvement observed in previous activation studies of autism [56,98].

Recapitulating our fcMRI results, in the control group we found many of the connectivity patterns for bilateral or unilateral seed volumes that were expected based on anatomical studies of caudate circuitry. The autism group showed functional connectivity in fewer of these expected areas known to participate in caudate-cortical circuits, such as the FEF and DLPFC. However, in direct group comparisons the autism group showed significantly more fcMRI effects for both bilateral and left caudate seeds, mostly in pericentral and premotor regions.

This latter finding may appear at odds with results from some previous fcMRI studies in autism [47,99]. Just et al [100], using ten pairs of cortical seed volumes, found reduced connectivity during sentence comprehension and hypothesized that the autistic brain was characterized by general "underconnectivity". It is possible that methodological differences account for the very different results in the present study, which was based on data acquired during visuomotor performance and which applied orthogonal regressors to remove effects of task-control cycles – contrary to methods implemented by Just and colleagues. However, Villalobos and colleagues [99], using very similar methods as those applied here to examine functional connectivity with primary visual area 17, also found predominantly reduced fcMRI effects during visuomotor coordination in autistic participants. The single most obvious methodological difference lies in the focus on cortico-cortical connectivity in previous studies that contrasts with the focus on *subcortico*-cortical connectivity in the present study. The pattern of results in the current limited fcMRI literature of autism would suggest that cortico-cortical functional connectivity tends to be reduced in autism – although in regionally and probably condition-specific ways [101] – whereas functional connectivity between subcortical nuclei, such as basal ganglia and thalamus [102], and cerebral cortex tends to be increased. Based on the current dataset, it cannot be determined whether partially increased fcMRI effects in our autism group may have been affected by non-specific states, such as general arousal (see discussion in ref. [102]). The potential impact of such states on low-frequency BOLD correlations is not established. Behavioral findings from our study showed that autistic participants were slightly faster than control subjects in condition A (index finger pressing), but this difference was not significant. In condition B (6-digit sequences), response times in the autism group were longer than in the control group, which appears inconsistent with increased arousal or impulsivity.

Since atypical or incompletely established hand preferences are common in autism [103,104], our study included three subjects who were either left-handed or ambidextrous. Although groups were matched for atypical handedness and although activation effects of visuomotor coordination compared to simple finger movement were not characterized by obvious functional asymmetries [56], the inclusion of left-handed subjects certainly complicates the interpretation of lateralizing effects in our fcMRI analysis. We addressed these issues in two ways: First, through additional analyses of right-handed subsamples; and secondly by focusing on bilateral caudate seed volumes (which were presumed to be less vulnerable to lateralizing effects), while examining effects for unilateral caudate seeds in addition.

**Figure 1:**
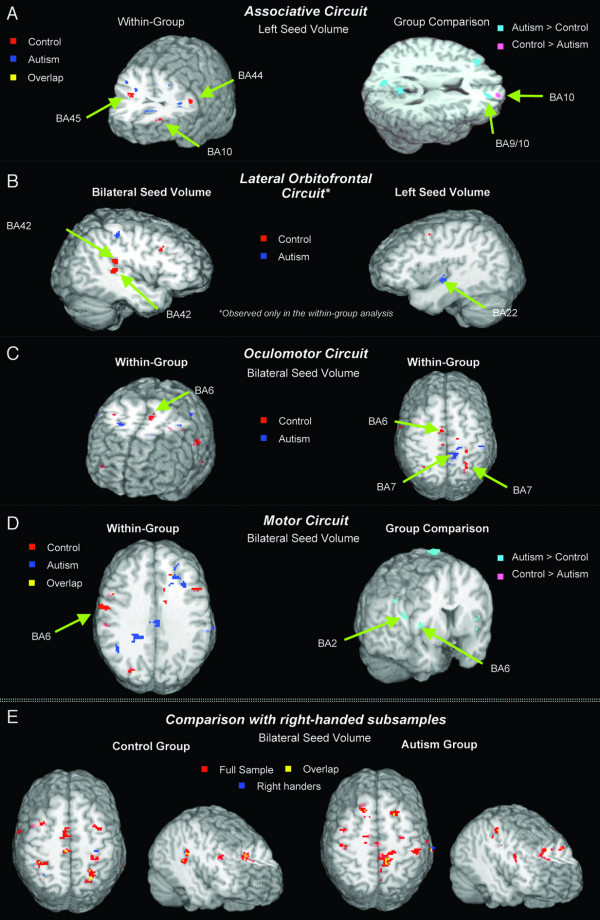
**Functional connectivity MRI effects**. Functional connectivity clusters for caudate seed volumes (*p *< .05, corr.), overlaid onto standard MRI renderings [108] and arranged by known anatomical networks with caudate participation. ***Associative circuit (A)***. In the control group, functional connectivity for left caudate seed volumes is seen in middle and inferior prefrontal cortex bilaterally, with a few small clusters of fcMRI effects for the autism group visible in neighboring frontal regions. Direct group comparison (right) shows greater fcMRI effects in controls in the frontopolar portions of area 10 in the right hemisphere, but inverse effects (autism > control) in more posterior portions on the border of areas 9 and 10. ***Lateral Orbitofrontal circuit (B)***. FcMRI effects associated with the lateral orbitofrontal circuit (LOF) are only seen in the within-group analyses. For the bilateral caudate seed, the control group shows fcMRI effects in the superior temporal gyrus. A corresponding effect in the autism group is only seen for the left caudate seed, with an fcMRI cluster extending from area 22 into the posterior insula. ***Oculomotor circuit (C)***. The control group shows fcMRI effects for the bilateral caudate seed in close vicinity of the frontal eye fields (as expected based on published stereotactic coordinates; see main text). FCMRI effects for the autism group are only seen in distal portions of area 6 and in area 7 (precuneus). ***Motor circuit(D)***. Within-group analysis for the bilateral caudate seed shows a single cluster for the control group in left premotor area 6. However, on direct group comparison, numerous clusters showing greater fcMRI effects in the autism group are seen in pericentral and premotor cortices. ***Comparison with right-handed subsamples (E)***. Comparison between analyses for full samples (*n *= 8) and right-handed subsamples (*n *= 5) in control (red) and autism groups (blue) and overlap (yellow), showing largely consistent effects. A threshold of *p *= .00025 (uncorr.) was chosen for best combined visibility of effects on both analyses.

First, results for right-handed subsamples were largely consistent with those for complete samples (see Table 1 ' [see additional file 1]' and Fig. 1E), although overall less robust, as to be expected due to lower statistical power. Secondly, results for bilateral caudate seed volumes were largely consistent with those for left caudate seeds. On both analyses, the autism group showed greater fcMRI effects in frontoparietal cortices, especially in pericentral sensorimotor areas. Results for the right caudate seed were partly divergent, in particular in temporal and occipital regions, where fcMRI effects were stronger in the control than in the autism group. In the activation analyses, the control group showed bilateral activation in the lentiform nucleus, while activity in the autism group was seen only in the left hemisphere, extending from the putamen into the caudate nucleus, and thus into the seed volume used for fcMRI analyses [56]. Although effects directly associated with the task-control cycle were regressed out in our fcMRI analyses, involvement of the left caudate nucleus in visuomotor coordination seen on activation analyses for the autism group may in part explain overall greater fcMRI effects in this group for bilateral and left (but not right) caudate seeds.

It should be noted that our results are based on a relatively small sample of 8 autistic subjects. Especially in view of the suspected biological heterogeneity within the autism spectrum, it therefore remains open to what extent our largely exploratory findings will apply to the general autistic population, including lower-functioning individuals.

## Conclusion

Our study used fcMRI to examine functional neural networks incorporating the caudate nuclei and to determine whether the functional connectivity would be compromised in autistic individuals. Our finding of in part atypically enhanced functional connectivity between caudate nuclei and other brain regions in autistic individuals may indicate more diffusely organized fiber tracts, possibly due to reduced synaptic pruning. Some authors have speculated that autism may be characterized by increased local, but reduced long-distance connectivity [46,105]. While this pattern would be mostly consistent with previous fcMRI studies of cortico-cortical connectivity, our results suggest that it may have to be modified with regard to subcortico-cortical connectivity. Different fcMRI patterns may relate to differences in maturational schedules between subcortico-cortical and cortico-cortical) fibers and to the interaction of these maturational schedules with atypical brain growth patterns in autism [106,107]. With regard to neurocognitive characteristics of autism, atypically diffuse functional connectivity between caudate nuclei and cerebral cortex is consistent with previous reports of structural anomalies of the basal ganglia associated with stereotyped behaviors and executive impairment.

## Competing interests

The author(s) declare that they have no competing interests.

## Authors' contributions

KCT was responsible for the fcMRI analysis design and writing of the manuscript; LF, DL, and JRMI carried out analyses and prepared the manuscript for publication; RAM was responsible for the initial design of the task conditions, carried out the data acquisition, and participated in the writing of the manuscript.

**Figure 2:**
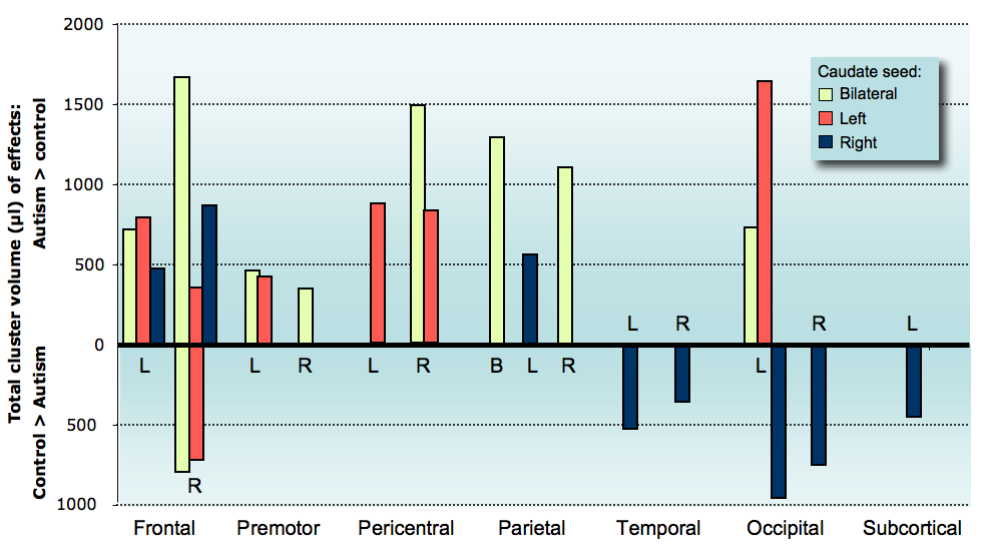
**Overview of direct group comparisons**. Each bar represents the total volume of effects (in μl) on group comparisons per subdivision of the brain (corresponding to the subdivisions used in Tables 1–3 ' [see additional file 1]'), shown separately for different seed volumes. Hemispheres of each brain subdivision are indicated by letters (L = left; R = right; B = bilateral). Upward bars show total fcMRI effects that were significantly stronger in the autism compared to the control group; downward bars show inverse effects (greater in control group). Note that for the right frontal lobe both upward and downward bars are shown because clusters of effects in both directions (autism > control; control > autism) were found in this part of the brain. No bars are shown for subdivisions without significant group differences.

## Supplementary Material

Additional File 1 fc caudate tables. The fcMRI data provided in this Excel file represent the regions of connectivity for each group (Autism and Control) referenced in the manuscript. The results detailed in Table 1 provide the regions of connectivity first for the full-sample, and then for the right-handed sub-sample found for the bilateral seed volume. Tables 2 and 3 detail the regions of connectivity seen in the full-sample for the left and right caudate seed volumes, respectively. Click here for file
